# Assessment of Pfs25 expressed from multiple soluble expression platforms for use as transmission-blocking vaccine candidates

**DOI:** 10.1186/s12936-016-1464-6

**Published:** 2016-08-11

**Authors:** Shwu-Maan Lee, Chia-Kuei Wu, Jordan Plieskatt, David H. McAdams, Kazutoyo Miura, Chris Ockenhouse, C. Richter King

**Affiliations:** 1 PATH Malaria Vaccine Initiative (MVI), 455 Massachusetts Avenue NW, Suite 1000, Washington, DC 20001-2621 USA; 2 PATH, 2201 Westlake Avenue, Suite 200, Seattle, WA 98121 USA; 3 Laboratory of Malaria and Vector Research, National Institute of Allergy and Infectious Diseases, National Institutes of Health, Rockville, MD 20852 USA

**Keywords:** Malaria, Pfs25, *Plasmodium falciparum*, Transmission blocking vaccine, Baculovirus, *Pichia*, Glycosylation, Recombinant protein

## Abstract

**Background:**

Transmission-blocking vaccines (TBVs) have become a focus of strategies to control and eventually eliminate malaria as they target the entry of sexual stage into the *Anopheles stephensi* mosquito thereby preventing transmission, an essential component of the parasite life cycle. Such vaccines are envisioned as complements to vaccines that target human infection, such as RTS,S as well as drug treatment, and vector control strategies. A number of conserved proteins, including Pfs25, have been identified as promising TBV targets in research or early stage development. Pfs25 is a 25 kDa protein of *Plasmodium falciparum* expressed on the surface of zygotes and ookinetes. Its complex tertiary structure, including numerous cysteines, has led to difficulties in the expression of a recombinant protein that is homogeneous, with proper conformation, and free of glycosylation, a phenomenon not found in native parasite machinery.

**Methods:**

While the expression and purification of Pfs25 in various systems, has been previously independently reported, here a parallel analysis of Pfs25 is presented to inform on the biochemical features of Pfs25 and their impact on functionality. Three scalable expression systems were used to express, purify, and evaluate Pfs25 both in vitro and in vivo, including the ability of each protein to produce functional antibodies through the standard membrane feeding assay.

**Results:**

Through numerous attempts, soluble, monomeric Pfs25 derived from *Escherichia coli* was not achieved, while *Pichiapastoris* presented Pfs25 as an inhomogeneous product with glycosylation. In comparison, baculovirus produced a pure, monomeric protein free of glycosylation. The glycosylation present for *Pichia* produced Pfs25, showed no notable decrease in the ability to elicit transmission reducing antibodies in functional evaluation, while a reduced and alkylated Pfs25 (derived from plant and used as a control) was found to have significantly decreased transmission reducing activity, emphasizing the importance of ensuring correct disulfide stabilized conformation during vaccine design and production.

**Conclusions:**

In this study, the biochemical features of Pfs25, produced from different expression systems, are described along with their impact on the ability of the protein to elicit functional antibodies. Pfs25 expressed using baculovirus and *Pichia* showed promise as candidates for vaccine development.

## Background

Malaria caused by *Plasmodium falciparum* is responsible for nearly a half million deaths annually, based on the estimates from the WHO [1]. The emergence of drug-resistant malaria strains over the last four decades has emphasized the desirability of the development of a safe and effective malaria vaccine. Vaccines play an important role in strategies for eliminating and eradicating malaria [2]. Particularly valuable would be a vaccine that blocks parasite function at multiple stages of the life cycle including transmission from humans to mosquitoes [3]. Such transmission-blocking vaccines (TBVs) would not block disease in the vaccine recipients directly but rather would reduce the prevalence of malaria in a population thereby complementing current vector control strategies and increasing the efficacy of the RTS,S vaccine which blocks infection from mosquito to human [4]. To advance such TBVs, the identification of appropriate target antigens, their expression, characterization, and preparation for experimental clinical testing is underway.

Malaria transmission requires transport of the *Plasmodium* parasite to the gut of the female *Anopheles stephensi* mosquito after feeding on an infected human. In the mosquito gut, the *Plasmodium* parasite undergoes sexual-stage development, replication, and invasion of the mosquito salivary glands leading to infectious sporozoites capable of infecting humans during the mosquito’s next blood meal [5]. As there are relatively few cells constituting the sexual stage in the mosquito, it has been proposed that vaccine induced neutralizing antibodies carried into the mosquito, as part of the blood meal, might be highly effective at halting the lifecycle of the *Plasmodium* parasite [5]. Several conserved proteins, specifically those involved in sexual-stage parasite development, have been identified as potential targets. Antibodies raised to these targets, have shown activity to inhibit laboratory-based assays of sexual stage parasite development thereby encouraging the advancement of candidate vaccines [6].

One of the primary targets for TBV development is the Pfs25 protein, an approximate 25 kDa sexual stage protein of *P. falciparum*, which is expressed on the surface of zygotes and ookinetes [6–8]. Pfs25 has a compact and unusual structure, which complicates its production and analysis [7, 9]. There are four-tandem epidermal growth factor (EGF)-like repeat motifs putatively anchored to the parasite surface by a glycosylphosphatidylinositol (GPI) moiety [10]. An additional complexity is added because *Plasmodium* parasites lack the N-linked glycosylation machinery, and Pfs25 contains multiple potential glycosylation sites that could then be aberrantly glycosylated when expressed in recombinant eukaryotic systems [11]. Whether this non-native glycosylation might affect functionality of Pfs25, especially as a TBV antigen, has not been comprehensively evaluated before in recombinant protein immunization. It seems likely that antibodies capable of interfering with Pfs25, will need to bind to the native configuration of the protein found on the parasite within the mosquito and that antibodies raised to a non-native protein might not be very active.

Immunogenicity of Pfs25 has been reported in both animals and in human clinical trials [12, 13]. The expression and purification of recombinant Pfs25 for these studies has been reported using different systems including yeast [11, 14–16], plant [17], *Escherichia coli* [18] and algae [19] along with delivery mechanisms for these reported proteins [20]. The objective was to compare these systems for the quality of Pfs25 obtained, including whether proper folding of the recombinant proteins occurs, and the impact protein folding has on the elicitation of functional antibodies. Three common expression systems (*E. coli*, *Pichia pastoris*, and baculovirus), which have been used for large-scale production of recombinant proteins, were used. Further, the purified proteins were evaluated using in vitro and in vivo tests using a previously reported plant-expressed Pfs25 [17] as an additional control antigen. This information is intended as a starting point for the selection of preferred production systems for vaccine generation and to inform the selection of the critical quality characteristics of the TBV candidate, Pfs25.

## Methods

### Constructs

The Pfs25 sequence from 3D7 clone (ACCESSION P13829) Ala 22 to Thr 193, lacking the native signal sequence and GPI-anchor was used to produce recombinant Pfs25 in *E. coli*, *Pichia*, and baculovirus. The GPI anchor sequence functions poorly in heterologous eukaryotic system and was not included [21]. Codon optimization for each expression system was performed by DNA2.0 with a C-terminal hexa-histidine tag added to the coding region to facilitate affinity purification.

#### *Escherichia coli*

Pfs25 with and without additional N-terminal periplasmic signal sequence (encoding MKYLLPTAAAGLLLLAAQPAMA of Pectate Lyase B of *Erwinia carotovora*) and with 5′ NdeI and 3′ XhoI restriction sites was cloned into pET41a (Novagen) via standard cloning procedures. Resulting plasmids denoted as pET41a-peri-Pfs25 (with periplasmic signal peptide) and pET41a-Pfs25 (without signal peptide), were sequenced and verified.

#### *Pichia*

Briefly, two mutations were introduced (N112Q and N187Q) to avoid N-glycosylation [17]. The resulting Pfs25 fragment with 5′ BamHI and 3′ EcoRI restriction sites was cloned into pPIC9K (Thermo Fisher) in frame with alpha-factor secretion signal. The expression plasmid denoted pMBL003-Pfs25 was sequenced and verified. pMBL003-Pfs25 was transformed into *Pichia pastoris* BICC9682, and cells plated on YNBD(Yeast Nitrogen Base Dextrose) agar plates at 30 °C for 3 days. Approximately 100 clones were screened for the expression of Pfs25 and an additional 2000 clones on G418 plates screened for multi-copy integrants.

#### Baculovirus

Synthetic pfs25 containing N112Q and N187Q mutations as described in *Pichia*, with an additional N-terminal secretion signal (MKFLVNVALVFMVVYISYIYAD from Honeybee Melittin) was cloned into pFastBac vector (Invitrogen) with BamHI (5′) and EcoRI (3′) sites and the resulting plasmid pFastBac-Pfs25 was sequence verified. The generation of recombinant virus followed Users’ Manual of Bac-to-Bac system (Invitrogen). Briefly, pFastBac-Pfs25 was transformed into *E. coli* DH10Bac to generate recombinant bacmid and colonies grown at 37 °C for 48 h on LB agar plates containing Tetracycline (10 μg/ml), kanamycin (50 μg/ml), gentamycin (7 μg/ml), IPTG (40 μg/ml) and X-Gal (5-bromo-4-chloro-3-indolyl-*β*-d-galactopyranoside, 100 μg/ml), according to users’ manual. The bacmids from selected colonies were confirmed by PCR and sequencing, then used to transfect Super Sf9 cells (Oxford Expression Technologies) for the generation of recombinant baculovirus stock (P1), using CellFECTIN II (Invitrogen) following Bac-to-Bac manual. P1 virus (approximately 14 ml) was harvested and stored at 4 °C protected from light. Two-milliliters of the P1 virus was used to amplify P2 baculovirus after infecting fresh super Sf9 cells at 27 °C for approximately 90 h and similarly to produce high titer P3 virus; remaining P1 baculovirus supernatant was stored at −80 °C. A P3 virus volume of 400 ml for further expression was harvested and titered using BacPAK titer kit (Clontech).

### *Escherichia coli* protein expression

Several methods to express soluble Pfs25 in *E. coli* were used including cell lines BL21 (DE3) and BLR(DE3), co-expression of protein disulfide bond isomerase (DsbC), expression in lower temperature (20 °C), and IPTG concentrations (1 and 0.25 mM). All of which did not yield sufficient quantity of soluble, monomeric Pfs25 for further purification. Briefly, pET41a-Pfs25 (cytoplasmic) and pET41a-peri-Pfs25 (periplasmic) plasmids were transformed in BL21 (DE3) and BLR (DE3), respectively, and clones were selected and verified. Expression induction at 37 and 20 °C was carried out for 3 and 16 h, respectively at an optical density A_600_ of 0.8. Induced cultures were harvested by centrifugation for 10 min at 4 °C. The cell pellet was collected and lysed using BugBuster Protein Extraction Reagent (Novagen) per the manufacturer’s instructions. Soluble and insoluble fractions were separated via centrifugation.

### *Pichia* protein expression and purification

Approximately 100 clones of pMBL003-Pfs25 in *Pichia pastoris* were screened for protein expression in 2 ml deep well plates with 2 ml complex medium containing 2 % dextrose for 2 days. Medium was then removed and replaced with 2 ml of complex medium with 1 % methanol for 3 days, with 1 % methanol feed once a day. Samples were collected on the third day post induction. Three high-yield clones were identified by analyses with reducing SDS-PAGE (Pfs25 expression band at 20 kDa), and evaluated with anti-His (Qiagen) or anti-Pfs25 mAb 4B7 [6, 22, 23] (BEI Resources) Western blots as described.

#### *Pichia* (G418 screening)

In parallel, clones were screened on YPD (Yeast peptone dextrose) agar in the presence of G418 (0.5, 1 and 2 mg/ml) in 96-well plates—corresponding to potential increased copies of gene of interest and hence, likely increased protein yield. G418 positive clones, including ten clones by 0.5 mg/ml G418, eight by 1 mg/ml G418, and two by 2 mg/ml G418 were subjected to small-scale expression as described earlier, which led to identification of slightly better protein expressers. A selection of ten clones was then evaluated in 100 ml BMGY (Buffered Glycerol complex medium) and induced with BMMY (Buffered Methanol complex Medium) using 1 % methanol feeding per day for 3 days followed by analysis (reducing SDS-PAGE). Two colonies, C6 and H4, produced similar yield and quality of Pfs25, and C6 was arbitrarily selected for 800 ml expression and further purification.

#### Purification

Supernatant from G418 (clone C6) was purified using Ni–NTA resin (Qiagen) using standard protocol. Briefly, 200 ml supernatant was adjusted to 1000 ml with 50 mM Tris–HCl (pH 8.0), 150 mM NaCl, applied to column and washed (10 CV, column volume) with 5 mM Imidazole in 50 mM Tris–HCl (pH 8.0), 150 mM NaCl. Proteins were eluted with 300 mM Imidazole in 50 mM Tris–HCl (pH 8.0), 150 mM NaCl, analysed, pooled, and dialyzed into PBS buffer, pH 7.4. The pool was concentrated and further purified by gel filtration (Superdex 75).

### Baculovirus expression and purification

Using Super Sf9 cells, three different MOI’s (multiplicity of infection, 1, 3, and 5) were screened at a 30 ml scale as well as uninfected cells (negative control). Post infection (48 and 72 h), 1 ml of culture was collected and centrifuged for 5 min. The supernatant was analysed via reducing and non-reducing SDS-PAGE and Western blot. Based on analysis of Pfs25 post infection of 72 h, no significant difference between the three MOI’s tested was seen and further expression proceeded with MOI = 1 at 100 ml and 72 h.

#### Purification

Supernatant was used for batch binding overnight with ~2 ml of Ni–NTA resin (Qiagen, Cat No: 30410) at 4 °C on table top rocker platform. Two washing steps were used: Wash buffer (1): 1x PBS with 10 mM Imidazole, pH 7.2 (10 CV) and wash buffer (2): 1x PBS with 20 mM Imidazole, pH 7.2 (10 CV). Protein was eluted via a step gradient with elution buffers (1): 1x PBS with 50 mM Imidazole, pH 7.2 and Elution buffer and (2): 1x PBS with 500 mM Imidazole, pH 7.2. Elution fractions were then analysed and those containing Pfs25 were pooled, concentrated, and further purified on Superdex 75 column.

### Plant control protein

Pfs25 derived from *Nicotiana benthamiana* was provided by Fraunhofer CMB (Newark, DE) and utilized as a control antigen. The soluble protein, termed Pfs25MF1E, and encompassing amino acid 23–193 of the Pfs25 sequence, with a theoretical mass of 20,021 daltons is fully described in [17]. The protein was provided at 1.65 mg/ml and in 20 mM Tris buffer containing 70 mM NaCl, pH 8.0 as previously described [17].

### SDS-PAGE

The samples were used with either (1) 4X non-reducing NuPAGE LDS (Lithium dodecyl sulfate) sample buffer (non-reducing and non-boiled) or with reducing agent and heated to 95 °C for 5 min or (2) 4X SDS sample buffer and boiled for 10 min at 99 °C (reducing and boiled). SDS-PAGE gels (4–12 % NuPAGE Bis–Tris or 15 % Tris–glycine) were loaded in a final volume of 20 μl/well and run at 150–200 V for 35–50 min in 1X MES or 1X SDS running buffer.

### Western blotting

Following SDS-PAGE, proteins were transferred onto PVDF membrane and blocked in 5 % skim milk (1X Tris buffered saline, TBS) at room temperature for 1 h. Primary antibodies were prepared at 1:5000 dilutions of Penta His antibody (Qiagen Cat No:- 34460), 1:2000 dilution of Anti-Pfs25 mAb 4B7 (BEI), or 1:1000 dilutions of 1G2 (final 2 µg/ml) in 1 % skim milk in 1X TBS buffer containing 0.05 % Tween-20 (TBS-T). Blots were incubated in the primary antibody solution for 1 h at room temperature or overnight at 2–8 °C. Membranes were washed with 1X TBS-T buffer (3X for 10 min) and secondary antibody 1:1000 dilution of goat anti mouse IgG-HRP (Santa Cruz) or 1:4000 goat anti mouse IgG Alkaline Phosphatase (BioRad) in 1 % skim milk (1X TBS-T buffer) was added and incubated at room temperature for 1 h. Membranes were then again washed with 1X TBS-T buffer (3X for 10 min). HRP labeled blots were developed with TMB (3,3,5,5′-Tetramethylbenzidine) substrate. Alkaline Phosphatase labeled blots were developed using Bio-Rad Alkaline Phosphatase Conjugate Substrate Kit.

### Kinetic endotoxin assay

Spectramax plus spectrophotometer (kinetic endotoxin assay) was used to quantify endotoxin content of purified Pfs25, with EndoSafe Endotoxin standard (Charles River), EndoSafe Lysate (Charles River), and EndoSafe LAL Reagent Water (Charles River).

### Parasite extract preparation

Mature gametocyte cultures were prepared as reported previously [24]. From the stage V gametocytes, zygote- and ookinete-stage parasites were induced in vitro using the method described by Ghosh et al. [25]. The parasite extract containing native Pfs25 was prepared by three times freeze-and-thaw of the *P. falciparum* NF54 gametocyte/zygote/ookinete parasites. The human serum and red blood cells used for the gametocyte cultures (extract preparation and SMFA) were purchased from Interstate Blood Bank (Memphis, TN).

### Size exclusion HPLC (SE-HPLC) analysis

SE-HPLC analysis for purified Pfs25 proteins from *Pichia* and baculovirus was performed on a BioAssist G3SWxl column (7.8 × 300 mm, TOSOH Biosciences, King of Prussia, PA) with a Shimadzu Prominence UFLC HPLC system at a flow rate of 0.7 ml/min in 0.2 M sodium phosphate pH 6.8. A gel filtration standard (Bio-Rad, Hercules, CA) was used in column calibration.

### Reversed-phase chromatography

Samples of Pfs25 were analysed using an Ultimate 3000 UHPLC system (Thermo Scientific) with a 2.6 µm, 2.1 × 150 mm C-18 column (Thermo Scientific) at a flow rate of 0.2 ml/min. C-18 column and auto-sampler temperatures were set at 60 and 5 °C, respectively. The elution of Pfs25 was monitored using absorbance (214 nm). Mobile phase (A) consisted of water with 0.1 % trifluoroacetic acid (TFA) and mobile phase (B) consisted of acetonitrile with 0.1 % TFA with a gradient of: 1 % B (5 min), 1–20 % B (2 min), 20–50 % B (30 min), 50–99 % B (2 min), 99 % B (3 min), 99–1 % B (2 min), and 1 % B (5 min). The Pfs25 samples were also run without column to determine sample recovery (82 ± 2 % for baculovirus protein and 70 ± 3 % recovery for *Pichia* protein).

### Free thiol determination

Free thiol (number of free cysteine residues) in each protein sample was measured using Measure iT free thiol assay kit (Life Technologies, Carlsbad, CA) following manufacturer’s instructions. Samples were diluted in either ultrapure water or 2 M guanidine-HCl and 50 μg of each Pfs25 sample was used for non-denaturing conditions and 25 μg of each Pfs25 sample was used for denaturing conditions. A standard curve (R^2^ = 0.983) was constructed using known concentrations of reduced glutathione. Fluorescence was measured using a SpectraMax M5 plate reader (Molecular Devices, Sunnyvale, CA).

### Intact mass spectrometry

Intact masses of each sample (in duplicate) were measured using a SYNAPT G2 hybrid quadrupole/ion mobility/TOF mass spectrometer (Waters Corporation, Milford, MA) with the assistance of the Mass Spectrometry and Analytical Proteomics Laboratory at the University of Kansas. The instrument was operated in a sensitivity mode with all lenses optimized on the MH + ion from the control Leucine Enkephalin and sample cone voltage of 40 eV. Argon was admitted to the trap cell operated at 4 eV for maximum transmission. Spectra were acquired at 9091 Hz pusher frequency covering the mass range from 100 to 3000 unified atomic mass unit and accumulating data for two seconds per cycle. Time to mass calibration was made with NaI cluster ions acquired under the same conditions. Mass spectra of [Glu^1^]-Fibrinopeptide B were acquired in parallel scans and doubly charged ions at m/z 785.8426 were used as a lock mass reference.

Samples were desalted via reversed phase PRP-1 column, 1 cm, 1 mm I.D. (Hamilton, 10 µm particles packed by hand) using a NanoAcquity chromatographic system (Waters Corporation) and solvents A (99.9 % H_2_O, 0.1 % formic acid) and B (99.9 % acetonitrile, 0.1 % formic acid) over a short gradient from 1 to 70 % B in 4 min with a flow rate of 20 µl/min. MassLynx 4.1 software (Waters Corporation) was used to collect data and deconvolute the protein spectra for molecular weight determination.

### Alkylation of Pfs25 and amino acid analysis

Pfs25 protein derived from plant at 1 mg/ml, pH 8.0 was reduced with 20 mM DTT at 60 °C for 30 min and alkylated with 40 mM iodoacetamide at 37 °C for 30 min in the dark; alkylation was quenched with large excess of β-mercaptoethanol. The extent of modified cysteines was analysed by amino acid analysis (AAA) at AI BioTech (Richmond, VA) with pre-column derivation using *O*-phthalaldehyde (OPA) and 9-fluorenylmethyl chloroformate (FMOC).

### Mouse immunization and SMFA

To produce anti-Pfs25 sera in CD-1 mice for SMFA, 10 µg of Pfs25 was formulated with ISA720 (Montanide) for each of the following groups: Group 1, baculovirus produced Pfs25; Group 2, *Pichia* produced Pfs25; Group 3, positive control (plant produced Pfs25 [17]); Group 4, negative control (reduced alkylated plant protein Pfs25) and Group 5, adjuvant only control. The groups of ten mice were injected (*intramuscularly*) on day 0 and 21. On day 42 sera were collected and individual sera were tested by ELISA. In addition, sera were pooled, IgG affinity purified and tested at 0.75 mg/ml as well as three-fold dilutions (0.250, 0.083 and 0.028 mg/ml) for SMFA activity [24]. Briefly, *P. falciparum* NF 54 gametocytes and purified IgG from mice immunized with Pfs25 were fed to female *Anopheles stephensi* mosquitoes. Mosquitoes were then dissected 8 days after the feeding and the number of oocysts counted to measure transmission reduction activity.

### ELISA

Basic methodology of ELISA has been described by Miura et al. [26]. All ELISA plates were coated with plant produced Pfs25 [17] at 100 ng/well. Based on a standard curve generated with a serially diluted ELISA reference standard (a pool of anti-Pfs25 antisera generated against a plant-produced Pfs25 VLP [27]), a relative antibody level (i.e., ELISA units) of test sample in the same plate was determined.

## Results and discussion

### Pfs25 expressed as soluble protein in *Pichia* and baculovirus

Recombinant His-tagged Pfs25 was produced as an intracellular protein in *E. coli*, and as secreted proteins in *Pichia* and baculovirus (super Sf9) at small scale and purified by Ni–NTA chromatography and gel filtration for subsequent evaluation. In *E. coli*, the majority of Pfs25 expressed was present in inclusion bodies and, therefore, sufficient soluble monomeric Pfs25 from *E. coli* lysates for analysis was not achievable.

It has been reported that it is possible to conduct refolding of Pfs25 [18] however this was not attempted in these studies in an effort to pursue a soluble secreted *E. coli* protein. Hereafter, Pfs25 solely from the *Pichia* and baculovirus expression systems were used for further biochemical in vitro and functional in vivo analysis. A well-characterized Pfs25, derived from plant [17], was provided and used as a control for these newly expressed Pfs25 recombinant proteins.

### Characterization of purified proteins

**Fig. 1:**
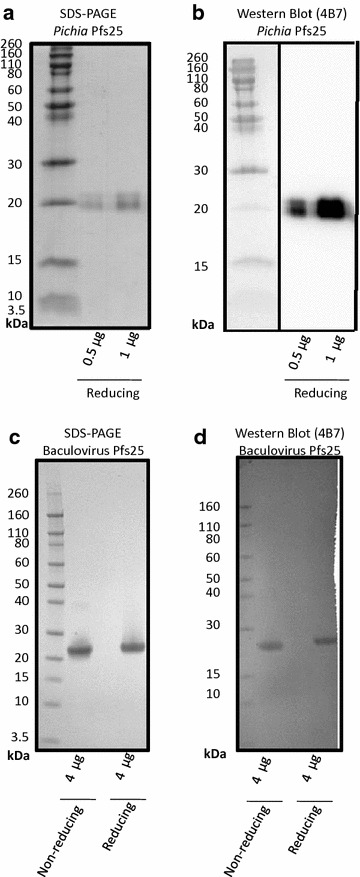
SDS-PAGE and Western blot (4B7) of *Pichia* and baculovirus Pfs25. **a** SDS-PAGE and **b** Western blot using 4B7 monoclonal antibody of purified Pfs25 from *Pichia* under reducing conditions (non-reduced not show). **c** SDS-PAGE and **d** Western blot using 4B7 monoclonal antibody of non-reduced and reduced purified Pfs25 from baculovirus

Pfs25 purified from *Pichia* gave rise to a major protein migrating at ~20 kDa and a minor band at ~21 kDa present at approximately 50 % of the intensity of 20 kDa band (Fig. 1a). The doublet corresponded to Pfs25 by western blotting using Pfs25 specific mAb 4B7 (Fig. 1b), which recognizes a β-hairpin epitope within the ILDTSNPVKT peptide sequence of the third EGF-like domain of native Pfs25 [22, 23]. Further investigation described below, using mass spectrometry, confirmed the doublet arose from protein glycosylation. For baculovirus expression, the purified Pfs25 protein presented as a single band of ~20 kDa and greater than 90 % purity (Fig. 1c), with identity confirmed with the 4B7 mAb (Fig. 1d).

**Fig. 2:**
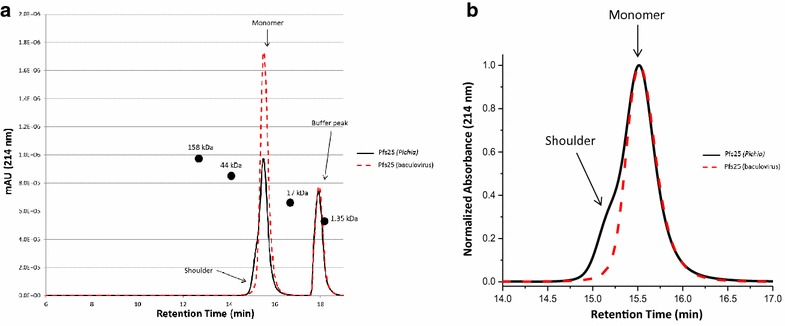
SE-HPLC of *Pichia* and baculovirus Pfs25. **a** Pfs25 from *Pichia* (*black line*) and baculovirus (*dashed red line*) eluted predominantly as a monomer. *Arrows* indicate shoulder observed for *Pichia* Pfs25 that most likely represents a higher molecular weight component co-eluting with monomer. The shoulder present for *Pichia* Pfs25 could not be fully resolved and integrated. Molecular weight gel filtration standards (Bio-Rad, Hercules, CA) indicated a monomer, with Pfs25 eluting at its expected molecular weight (between 44 and 17 kDa). **b** Normalized absorbance for eluted monomer peaks

In addition, the proteins were evaluated utilizing SE-HPLC to determine the aggregation state of the purified proteins. The *Pichia* and baculovirus proteins both eluted primarily as a monomer and contained only ~0.2 % of higher molecular weight species. It should be noted, the *Pichia* Pfs25 had a front shoulder (Fig. 2) that most likely represents a slightly larger molecular weight species or heterogeneity of the preparation (see intact MS analysis, Fig. 4a later) that is co-eluted with the monomer fraction (Fig. 2).

### Demonstration of conformational epitopes bound by 1G2 mAb

**Fig. 3:**
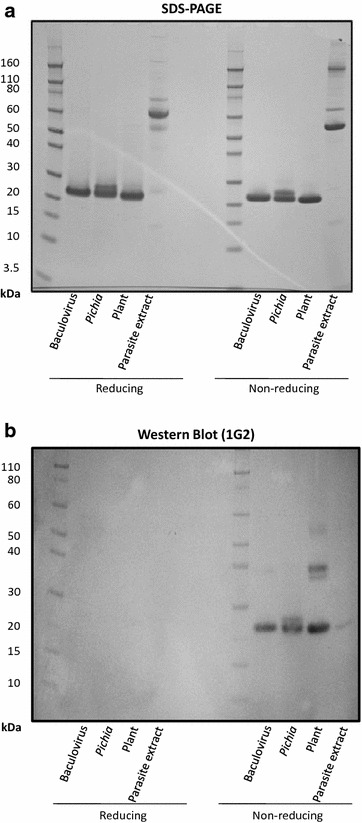
SDS-PAGE and Western blot using a conformational antibody of various Pfs25 proteins and native parasite extract. **a** SDS-PAGE, **b** Western blot using the conformational monoclonal antibody 1G2

To further assess the conformation of the purified Pfs25 from both expression systems the transmission-blocking monoclonal antibody 1G2, which can efficiently block the growth of parasites in mosquito vectors [28], was used for Western blotting analysis. A third recombinant Pfs25 expressed in plants [17], along with whole parasite lysates containing native Pfs25, were included for comparison with the recombinant Pfs25 (Fig. 3) prepared under this study. This data indicates that the 1G2 antibody recognized reduction sensitive, conformation dependent functional epitopes in both the recombinant and native Pfs25 derived from whole parasite lysates (Fig. 3). A Ponceau S control staining of the blot confirmed the transfer of proteins both in non-reduced and reduced form to the blotting membrane. The results provided evidence that Pfs25 produced from the *Pichia* and baculovirus contained a conformation recognized by this functional and conformation-dependent monoclonal antibody. It should be further noted, while the plant expressed Pfs25, contains a Glycine to Alanine substitution at aa131 [17, 27], there was no apparent impact on the binding via 1G2 mAb by this Western analysis.

### Glycosylation associated with *Pichia* Pfs25

**Fig. 4:**
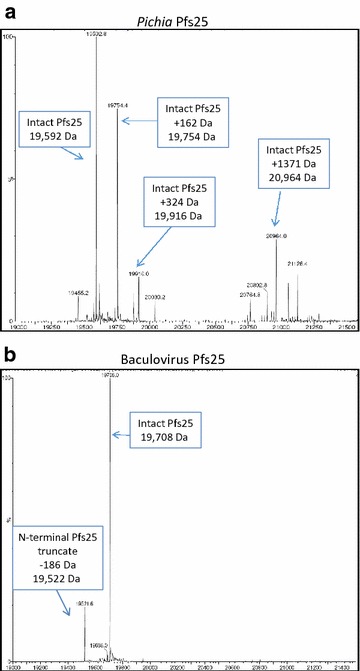
Intact mass spectrometry analysis of recombinant Pfs25 proteins. Intact mass spectrometry analysis of **a** Pfs25 derived from *Pichia* and **b** Pfs25 expressed from baculovirus. Mass determinations are indicated in *arrow boxes*, with the addition of mass adduct noted from the parent, predominant mass

Efforts to overcome post-translational modifications involving glycosylation have been mostly restricted to mutation of putative N-linked glycosylation sites [11, 29]. In the constructs utilized here, two putative glycosylation sites were mutated from N to Q (positions 112 and 187). In light of this, SDS-PAGE revealed Pfs25 from *Pichia* presented two predominant bands (Figs. 1, 3) with a relative intensity of 1:2 (21 kDa:20 kDa) based on densitometry. To further discern the nature of the doublets in *Pichia*-expressed Pfs25, intact mass spectrometry was performed to determine if the higher molecular band present on SDS-PAGE was indeed a result of glycosylation. The results from mass spectrometry indicated that *Pichia* Pfs25 contained added sugar adducts, as evidenced by the mass difference of 162 and 324 Daltons (Fig. 4a), the expected mass difference due to the presence of sugar moieties. Pfs25 produced in *Pichia* has been previously reported with *O*-glycosylation [11], where mannose residues, typical of *P. pastoris* [30, 31], were identified. It should be noted that while previously three possible *N*-linked glycosylation sites were mutated [11], in the studies reported here, only two such sites were mutated. The decision to mutate only two sites was based on historical literature [17] as well as a predictive analysis using NetNGlyc 1.0 Server [32] and GlycoEP [33]; as the asparagine at position 165 had a consistent low probability (<50 %) of potential glycosylation. In comparison, the baculovirus expressed Pfs25 presented as a single predominant band and >90 % pure based on SDS-PAGE densitometry indicating absence of glycosylation.

As shown in the same intact mass analysis (Fig. 4a; Table 1) the *Pichia* Pfs25 mass matched the oxidized form of Pfs25 (19,592.5 Da), which implicated the presence of 11 pairs of disulfides [14]. The intact mass analysis of baculovirus Pfs25 (Fig. 4b; Table 1) matched the predicted molecular mass of Pfs25, consistent with the absence of glycosylation with all cysteines in the oxidized form. A minor species 186 Da lower than the main species (Fig. 4b) was however present in this baculovirus preparation, and N-terminal sequencing showed that this minor species was a N-terminal truncate of the first two amino acids (D-A). Such truncation is consistent with the probability of signal peptide cleavage sites as predicted by SignalP [34]. This truncated form was present at approximately 24 % the full-length protein based on a semi-quantitative estimate from N-terminal sequencing data by Edman degradation [35].

**Table 1:** Physical properties of recombinant Pfs25

	Theoretical mass (Da) R-SH	Theoretical mass R-S-S-R (Da)	Observed mass (Da)	Difference^a^ (Da)	N-terminal sequence
*Pichia*	19,614.5	19,592.5	19,592.8 ± 0.5	0.3	AKVTV
Baculovirus	19,729.6	19,707.6	19,708.0 ± 0.5	0.4	DAKVT

^a^Difference between observed mass and oxidized form theoretical mass

### Cysteine oxidization and analysis of homogeneity

To confirm that recombinant Pfs25 proteins were indeed in the oxidized form, the number of thiol groups exposed on the protein surface with and without 2 M guanidine HCl was measured. Both proteins (*Pichia* and baculovirus) contained <0.1 % free thiol, as no fluorescence signal was detected above the buffer control, supporting the observations that all 22 cysteines were oxidized and disulfide paired as suggested by the intact mass observations described above (Fig. 4). Further, the Plant Pfs25 protein used as a control and comparator throughout these studies, was also tested and confirmed to contain <0.1 % free thiol.

**Fig. 5:**
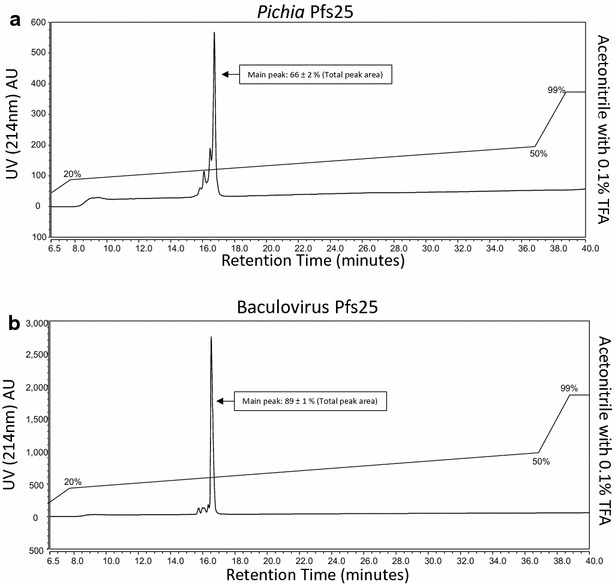
Reverse phase HPLC analysis of recombinant Pfs25 proteins. Reverse phase HPLC of **a** *Pichia* derived Pfs25 and **b** Baculovirus derived Pfs25

A reverse phase HPLC (RP-HPLC) assay was utilized to further quantify the inhomogeneity (Fig. 5) of the *Pichia* produced Pfs25 in comparison to the baculovirus product. The analysis utilizing this RP-HPLC method showed that the *Pichia* produced Pfs25 was approximately 66 % pure (Fig. 5a), whereas in contrast the baculovirus produced Pfs25 appeared to be of 89 % purity (Fig. 5b).

### Elicitation of functional antibodies

After observing glycosylation in *Pichia*-expressed Pfs25, whether this modification would affect the biological activity of Pfs25, was investigated. While a previous study utilizing DNA vaccination showed limited detrimental effects of glycosylation on transmission reducing activity [29], data on the administration of glycosylated recombinant antigens was limited. It is reasoned that glycosylation could change the overall protein conformation or occlude sites that may elicit functional antibody responses. To answer this, polyclonal antibodies were raised in mice to the various recombinant Pfs25 and the transmission reducing activity of the antisera measured by the reduction of oocysts in mosquito midguts using a standard membrane feeding assay [24]. Before immunization, it was confirmed that all purified proteins contained <2 EU/mg of endotoxin.

For the immunizations, the plant protein was used as a positive control and a reduced alkylated form of the plant protein was used as a negative control to confirm the importance of disulfide bond formation of Pfs25. Further analysis of the reduced alkylated Pfs25 preparation via amino acid composition analysis recovered 13 carboxymethylated cysteine, indicating at least 13 out of 22 cysteines were modified by alkylation.

**Fig. 6:**
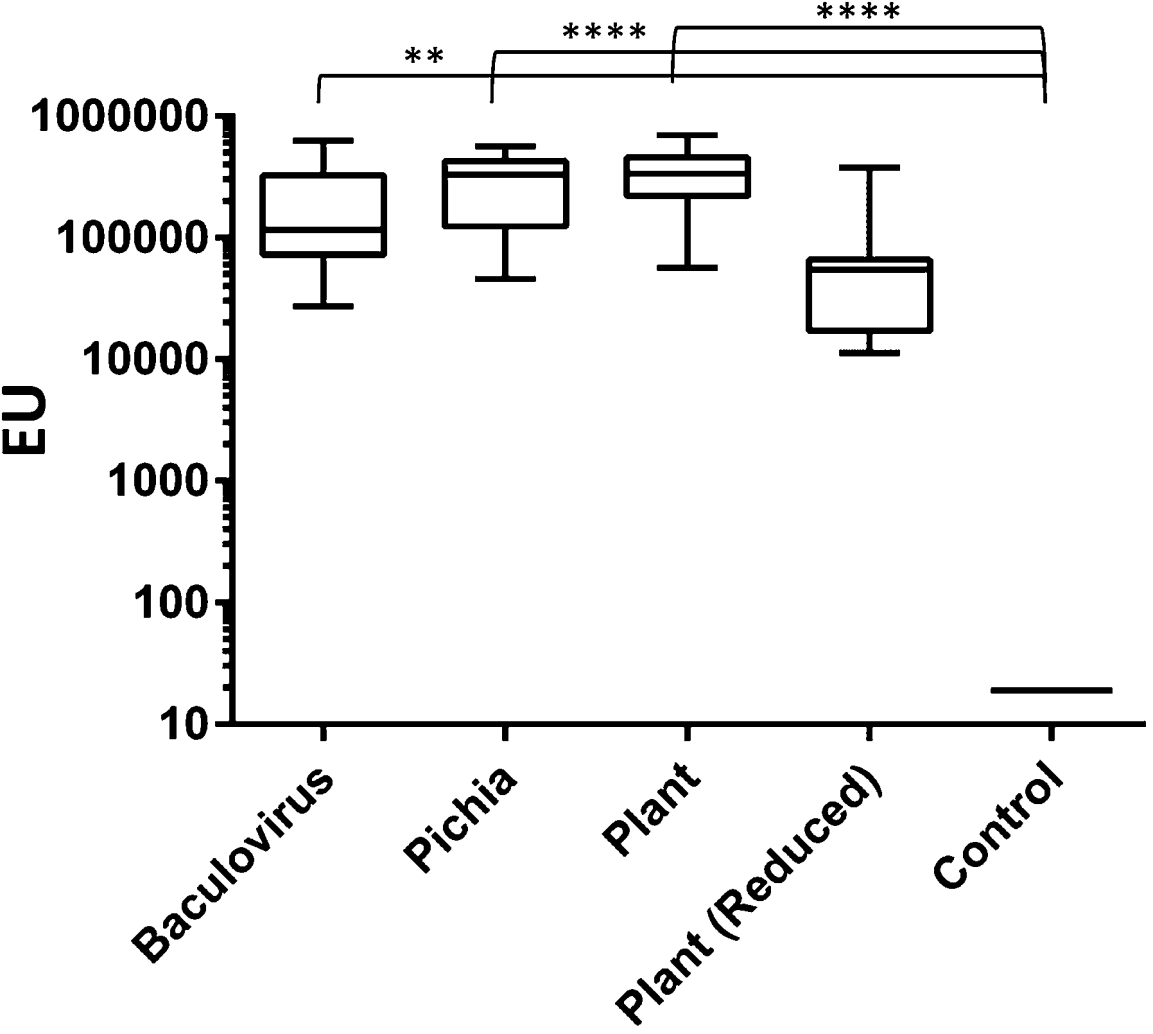
ELISA titer of individual CD-1 mice immunized with Pfs25. *Box whisker plot* of ELISA titer of CD-1 mice immunized with Pfs25 and controls presented in log scale of ELISA Units (EU). All serum samples were tested against plant Pfs25, not against immunogens. Line denotes median value for group, box shows 25–75 % interval and whiskers show min and maximum values (****p value <0.0001, **p value <0.01)

The antisera raised in mice were evaluated by ELISA for antibody titers and their functional activities were evaluated by SMFA. The ELISA titers of mouse sera raised by Pfs25 proteins of different expression systems (Groups 1–3) were not statistically different between the Pfs25 groups as determined through a non-parametric Kruskal–Wallis test and Dunn’s multiple comparisons test (Fig. 6). Statistical significance was determined, however, between each of the Pfs25 non-reduced groups (Groups 1, 2 or 3) and the control (Group 5) with p values <0.01 (Fig. 6). To probe for the elicitation of functional antibodies, the reduction of oocyst density was measured in an SMFA (Table 2). The results were consistent with previous observations of the Pfs25 immunogenicity [17] with each protein preparation demonstrating the ability to elicit transmission reducing activity >99 % at high concentrations of antibody. Purified IgG at a concentration of 750 μg/ml (Experiment 1, Table 2), elicited by non-reduced Pfs25 proteins did not reveal differences between different methods of preparation, and thus was further tested at threefold dilutions (Experiment 2, Table 2). Even at the lowest concentration tested (28 μg/ml), no statistically significant difference was observed among non-reduced Pfs25 groups (Groups 1–3) with all transmission reducing activity ≥98 %. Therefore, in *Pichia* expressed Pfs25 (Group 2), glycosylation did not apparently affect the transmission reducing activity as detected here, with results comparable to Pfs25 produced from baculovirus (Group 1) or plant (Group 3) that each exhibited little or no glycosylation (Table 2). In Group 4, where disulfides were disrupted, the sample produced lower antibody titers (Fig. 6) and yielded negligible transmission reducing activity (Table 2). This experiment confirmed that the conformation of Pfs25 held by disulfide bonds was essential to generate transmission reducing antibodies in mice, whereas glycosylation may not be as much of a concern for functional activity as previously thought. Moreover, Pfs25 produced from *Pichia* and baculovirus are active in raising functional antibodies that convey transmission reducing activity.

**Table 2:** Functional evaluation of antibodies against Baculovirus, *Pichia* and plant Pfs25 proteins

Sample	Conc^a^	Mean Ooc^b^	% inhibition^c^	p value^d^
Experiment 1				
Baculovirus	750	0.1 (0, 1)	100 (99, 100)	<0.001
*Pichia*	750	0 (0, 0)	100 (100, 100)	<0.001
Plant	750	0 (0, 0)	100 (100, 100)	<0.001
Plant (reduced)	750	25.1 (3, 45)	3 (−176, 67)	0.945
Control	750	25.8 (0, 58)	N/A	N/A
Experiment 2				
Baculovirus	250	0 (0, 0)	100 (100, 100)	<0.001
	83	0 (0, 0)	100 (100, 100)	<0.001
	28	0.4 (0, 2)	98 (94, 100)	<0.001
*Pichia*	250	0 (0, 0)	100 (100, 100)	<0.001
	83	0 (0, 0)	100 (100, 100)	<0.001
	28	0.3 (0, 2)	99 (95, 100)	<0.001
Plant	250	0 (0, 0)	100 (100, 100)	<0.001
	83	0 (0, 0)	100 (100, 100)	<0.001
	28	0.1 (0, 2)	100 (99, 100)	<0.001
Control	750	17.1 (0, 62)	N/A	N/A

Groups of ten mice were injected with 10 µg of Pfs25 adjuvanted with montanide on days 0 and 21. On day 42 sera were pooled, and the purified IgGs were tested at the indicated concentrations by SMFA

^a^IgG concentration (μg/ml) in a feeder

^b^Arithmetic mean (range) of oocysts intensity from 20 mosquitoes

^c^Percent inhibition of mean oocyst intensity and the 95 % confidence interval (95 % CI)

^d^Two-sided p values for testing whether % inhibition is significantly different from 0

## Conclusions

In the search for a transmission blocking vaccine based on Pfs25, the malaria community has been guided by the concept that an immunogen should mimic as much as possible the Pfs25 found on the surface of the parasite. In the current studies, Pfs25 was expressed, purified, and characterized in the *Pichia* and baculovirus systems. In the *E. coli* system, in spite of numerous attempts, soluble, monomeric Pfs25 was not achieved. While it may be possible to generate native configuration Pfs25 using refolding methods from the generated inclusion bodies [18], this was not pursued given the promising nature of results from the other expression systems. In contrast, both *Pichia* and baculovirus expression resulted in proteins with many characteristics consistent with the expected structure of the native Pfs25. In *Pichia,* the expressed Pfs25 was likely glycosylated, despite mutations of probable N-linked sites, and consequently resulted in multiple molecular weight forms. The baculovirus expressed Pfs25 presented a non-glycosylated and homogeneous recombinant protein. Through multiple methods, including the free thiol assay and intact mass analysis, both *Pichia* and baculovirus produced Pfs25 proteins contained 11 pairs of disulfides as expected. Two transmission blocking monoclonal antibodies 4B7 [22, 23] and 1G2 [28], also bind well to both Baculovirus and *Pichia* produced Pfs25 protein suggesting proper folding of the purified proteins.

The ability to elicit functional transmission blocking antibodies is the most important characteristic of any protein preparation to be considered for the generation of a vaccine. Given that *P. falciparum* apparently lacks such modifications, glycosylation has been a concern in the development of recombinant antigens for use as TBV and other malaria vaccine candidates. In this study, the evidence of limited glycosylation present in *Pichia* produced Pfs25 did not appear to diminish the ability to elicit transmission blocking antibodies and was consistent with findings carried out with Pfs25 DNA vaccines evaluating glycosylation of Pfs25 [29]. It is important, however, to recognize the limitations of SMFA testing, as it may not detect subtle differences in potency. It may also be possible to separate the various forms of Pfs25 (glycosylated and non-glycosylated) and in conjunction with a dose ranging study (versus a single 10 µg) to better determine the efficacy of a glycosylated Pfs25 as compared to non-glycosylated forms. As potent transmission blocking antibodies were elicited by the aglycosylated, homogeneous and stable Pfs25 produced from the baculovirus, this production system may be attractive for further vaccine development.

Lastly, the status of the disulfide bonds formed in the recombinant proteins was investigated. Proteins produced in *Pichia*, baculorvirus as well as the control plant produced protein showed evidence of no free cysteines by two methods, free thiol and mass spectrometric analysis. When these disulfide bonds are disrupted by reduction and alkylation the elicitation of functional antibodies (as measured by SMFA) was diminished, demonstrating that at least some of the disulfide bonds are essential for the function of the Pfs25 protein as an immunogen. Taken together, these results indicate that both *Pichiapastoris* and baculovirus expression systems have potential for the production of Pfs25 based transmission-blocking vaccines.

## References

[1] WHO (2015). World Malaria Report 2015.

[2] Birkett AJ (2015). Building an effective malaria vaccine pipeline to address global needs. Vaccine..

[3] Nunes JK, Woods C, Carter T, Raphael T, Morin MJ, Diallo D (2014). Development of a transmission-blocking malaria vaccine: progress, challenges, and the path forward. Vaccine..

[4] Birkett AJ, Moorthy VS, Loucq C, Chitnis CE, Kaslow DC (2013). Malaria vaccine R&D in the Decade of Vaccines: breakthroughs, challenges and opportunities. Vaccine..

[5] Wu Y, Sinden RE, Churcher TS, Tsuboi T, Yusibov V (2015). Development of malaria transmission-blocking vaccines: from concept to product. Adv Parasitol.

[6] Barr PJ, Green KM, Gibson HL, Bathurst IC, Quakyi IA, Kaslow DC (1991). Recombinant Pfs25 protein of *Plasmodium falciparum* elicits malaria transmission-blocking immunity in experimental animals. J Exp Med.

[7] Kaslow DC, Syin C, McCutchan TF, Miller LH (1989). Comparison of the primary structure of the 25 kDa ookinete surface antigens of *Plasmodium falciparum* and *Plasmodium gallinaceum* reveal six conserved regions. Mol Biochem Parasitol.

[8] Vermeulen AN, Ponnudurai T, Beckers PJ, Verhave JP, Smits MA, Meuwissen JH (1985). Sequential expression of antigens on sexual stages of *Plasmodium falciparum* accessible to transmission-blocking antibodies in the mosquito. J Exp Med.

[9] Kaslow DC, Bathurst IC, Lensen T, Ponnudurai T, Barr PJ, Keister DB (1994). *Saccharomyces cerevisiae r*ecombinant Pfs25 adsorbed to alum elicits antibodies that block transmission of *Plasmodium falciparum*. Infect Immun.

[10] Kaslow DC, Quakyi IA, Syin C, Raum MG, Keister DB, Coligan JE (1988). A vaccine candidate from the sexual stage of human malaria that contains EGF-like domains. Nature.

[11] Tsai CW, Duggan PF, Shimp RL, Miller LH, Narum DL (2006). Overproduction of *Pichia pastoris* or *Plasmodium falciparum* protein disulfide isomerase affects expression, folding and O-linked glycosylation of a malaria vaccine candidate expressed in *P. pastoris*. J Biotechnol.

[12] Draper SJ, Angov E, Horii T, Miller LH, Srinivasan P, Theisen M (2015). Recent advances in recombinant protein-based malaria vaccines. Vaccine..

[13] Wu Y, Ellis RD, Shaffer D, Fontes E, Malkin EM, Mahanty S (2008). Phase 1 trial of malaria transmission blocking vaccine candidates Pfs25 and Pvs25 formulated with montanide ISA 51. PLoS One.

[14] Zou L, Miles AP, Wang J, Stowers AW (2003). Expression of malaria transmission-blocking vaccine antigen Pfs25 in *Pichia pastoris* for use in human clinical trials. Vaccine..

[15] Shimp RL, Rowe C, Reiter K, Chen B, Nguyen V, Aebig J (2013). Development of a Pfs25-EPA malaria transmission blocking vaccine as a chemically conjugated nanoparticle. Vaccine..

[16] Kubler-Kielb J, Majadly F, Wu Y, Narum DL, Guo C, Miller LH (2007). Long-lasting and transmission-blocking activity of antibodies to *Plasmodium falciparum* elicited in mice by protein conjugates of Pfs25. Proc Natl Acad Sci USA.

[17] Farrance CE, Chichester JA, Musiychuk K, Shamloul M, Rhee A, Manceva SD (2011). Antibodies to plant-produced *Plasmodium falciparum* sexual stage protein Pfs25 exhibit transmission blocking activity. Hum Vaccin..

[18] Kumar R, Angov E, Kumar N (2014). Potent malaria transmission-blocking antibody responses elicited by *Plasmodium falciparum* Pfs25 expressed in Escherichia coli after successful protein refolding. Infect Immun.

[19] Gregory JA, Li F, Tomosada LM, Cox CJ, Topol AB, Vinetz JM (2012). Algae-produced Pfs25 elicits antibodies that inhibit malaria transmission. PLoS One.

[20] Jones DS, Rowe CG, Chen B, Reiter K, Rausch KM, Narum DL (2016). A Method for producing protein nanoparticles with applications in vaccines. PLoS One.

[21] Scheiblhofer S, Chen D, Weiss R, Khan F, Mostböck S, Fegeding K (2001). Removal of the circumsporozoite protein (CSP) glycosylphosphatidylinositol signal sequence from a CSP DNA vaccine enhances induction of CSP-specific Th2 type immune responses and improves protection against malaria infection. Eur J Immunol.

[22] Stura EA, Kang AS, Stefanko RS, Calvo JC, Kaslow DC, Satterthwait AC (1994). Crystallization, sequence and preliminary crystallographic data for transmission-blocking anti-malaria Fab 4B7 with cyclic peptides from the Pfs25 protein of *P. falciparum*. Acta Crystallogr D Biol Crystallogr.

[23] Stura EA, Satterthwait AC, Calvo JC, Stefanko RS, Langeveld JP, Kaslow DC (1994). Crystallization of an intact monoclonal antibody (4B7) against *Plasmodium falciparum* malaria with peptides from the Pfs25 protein antigen. Acta Crystallogr D Biol Crystallogr.

[24] Miura K, Deng B, Tullo G, Diouf A, Moretz SE, Locke E (2013). Qualification of standard membrane-feeding assay with *Plasmodium falciparum* malaria and potential improvements for future assays. PLoS One.

[25] Ghosh AK, Dinglasan RR, Ikadai H, Jacobs-Lorena M (2010). An improved method for the in vitro differentiation of *Plasmodium falciparum* gametocytes into ookinetes. Malar J..

[26] Miura K, Orcutt AC, Muratova OV, Miller LH, Saul A, Long CA (2008). Development and characterization of a standardized ELISA including a reference serum on each plate to detect antibodies induced by experimental malaria vaccines. Vaccine..

[27] Jones RM, Chichester JA, Mett V, Jaje J, Tottey S, Manceva S (2013). A plant-produced Pfs25 VLP malaria vaccine candidate induces persistent transmission blocking antibodies against *Plasmodium falciparum* in immunized mice. PLoS One.

[28] Cheru L, Wu Y, Diouf A, Moretz SE, Muratova OV, Song G (2010). The IC(50) of anti-Pfs25 antibody in membrane-feeding assay varies among species. Vaccine..

[29] Datta D, Bansal GP, Kumar R, Ellefsen B, Hannaman D, Kumar N (2015). Evaluation of the impact of codon optimization and N-linked glycosylation on functional immunogenicity of Pfs25 DNA vaccines delivered by In vivo electroporation in preclinical studies in mice. Clin Vaccine Immunol.

[30] Macauley-Patrick S, Fazenda ML, McNeil B, Harvey LM (2005). Heterologous protein production using the *Pichia pastoris* expression system. Yeast.

[31] Gemmill TR, Trimble RB (1999). Overview of N- and O-linked oligosaccharide structures found in various yeast species. Biochim Biophys Acta.

[32] Blom N, Sicheritz-Pontén T, Gupta R, Gammeltoft S, Brunak S (2004). Prediction of post-translational glycosylation and phosphorylation of proteins from the amino acid sequence. Proteomics.

[33] Chauhan JS, Rao A, Raghava GP (2013). In silico platform for prediction of N-, O- and C-glycosites in eukaryotic protein sequences. PLoS One.

[34] Petersen TN, Brunak S, von Heijne G, Nielsen H (2011). SignalP 4.0: discriminating signal peptides from transmembrane regions. Nat Methods.

[35] Niall HD (1973). Automated Edman degradation: the protein sequenator. Methods Enzymol.

